# How to deal with the unmotivated medical student in small group sessions?

**DOI:** 10.15694/mep.2017.000086

**Published:** 2017-05-19

**Authors:** Nienke M.J. Hensen, Olle ten Cate

**Affiliations:** 1 UMC Utrecht

**Keywords:** Small Group Teaching, Self-Determination Theory

## Abstract

This article was migrated. The article was marked as recommended.

**Introduction:** To increase the motivation of students at small group seminar education sessions, teachers and institutions often revert to rewarding the prepared students and/or punishing those who did not prepare. How effective is that? We sought to find theoretical claims or disclaims for this policy from Self-Determination Theory, which is an important contemporary theory about motivation. SDT distinguishes intrinsic and extrinsic motivation and provides evidence for the use of rewards and punishments.

**Aim:** The primary aim was to explore the effects of extrinsic rewards and negative incentives on the intrinsic motivation in the literature. A secondary goal was to provide practical tips for teachers to improve the motivation of medical students.

**Results:** Verbal rewards can increase the intrinsic motivation. Unexpected tangible and task-non-contingent tangible rewards appear to have no detrimental effect on the intrinsic motivation. All other expected tangible rewards and negative incentives, like threats and deadlines, have been found to undermine the intrinsic motivation. Autonomous self-regulated learning (intrinsic motivation, identified regulation and/or integrated regulation) is associated with high quality learning and well-being. Autonomous self-regulated learning is therefore the desired drive for learning and can be supported by a teacher via satisfying the needs for autonomy, competence and relatedness.

**Conclusion:** Extrinsic rewards and negative incentives should be avoided as they both undermine the intrinsic motivation. Autonomous self-regulated learning leads to more effective learning. Several practical tips that support one of the three basic psychological needs are discussed. Most are relatively easy to apply and stimulate autonomous self-regulated learning.

## Introduction

‘A teacher enters the classroom for a sessions with 11 students. He is excited, because the subject of the lesson is traumatology. His favorite topic. He has spent 3 hours preparing clinical cases. Students enter the classroom just before the lesson starts. Two of them come in 15 minutes late. During the lesson, the teacher tries to involve the students in solving the problems. Despite all his effort, the students barely respond. They haven’t prepared the questions and only give answers that are read from a purchased summary. There is little interaction. Most of the students wait for the teacher to explain the questions. Some of the students don’t pay any attention at all. After the lesson, the teacher leaves the room frustrated and grumpy.’

Every teacher may recognize this scenario. Unmotivated and unprepared students can substantially decline your enthusiasm and excitement ( van den Berg, Bakker, & Ten Cate, 2013). Teaching students who are motivated and well prepared is much more interesting. During these lessons there is more interaction, leading to more, and more thorough, discussions. It enables reaching the desired depth of the subject during the lesson instead of only highlighting the surface. What teachers regularly do in education to increase the motivation of students is reward the well prepared students and punish those who did not prepare. ( Deci, Koestner, & Ryan, 2001; Lyness, Lurie, Ward, Mooney, & Rambert, 2013) This can be done by grades for participation, best student awards, sending non-participating students out of the classroom, making classes mandatory, and requiring a mandatory e-module as preparation.

The question for this review is: What will actually lead to more motivated students?

Self-Determination Theory (SDT) was developed by the researchers Edward Deci and Richard Ryan ( Deci & Ryan, 1985) and is one of the major theories about motivation. It distinguishes two major types of motivation: intrinsic and extrinsic motivation. Intrinsic motivation refers to doing something out of one’s own interest. Extrinsic motivation refers to doing something because of extrinsic factors. ( Legault, 2016; Ryan & Deci, 2000a) The aim of this paper was to study the effect of extrinsic rewards and negative incentives, like deadlines, withholding marks or low marks after insufficient preparation or attendance, on the intrinsic motivation for small group education in medicine. The second aim was to provide practical tips for teachers to increase the motivation of medical students.

## Methods

A literature search was performed using Google Scholar as the primary search engine. Several combinations of the following search terms were used: intrinsic motivation; extrinsic motivation; self-determination theory or SDT; stimulate, increase, improve or support; reward(s); medical student(s); autonomy; threats; deadlines; competition. The references of relevant articles and references at the SDT website were screened for additional useful articles.(Ryan, Deci, & Hoefen, n.d.)

In the past 30-40 years much has been written about intrinsic motivation, extrinsic motivation and SDT. Therefore, only the most relevant articles were used for this paper. Studies were not used if they were not written in English or Dutch or in case the full text was not available.

## Results

### Intrinsic motivation

Motivation is defined as ‘a reason or reasons for acting or behaving in a particular way’ or ‘desire or willingness to do something’.(Motivation [Def. 1], n.d.) The *level* of motivation can vary and is dependent of many factors. As mentioned in the introduction, Deci and Ryan distinguish two types of motivation in SDT. ( Deci & Ryan, 1985; Ryan & Deci, 2000a) The most basic distinction is between intrinsic and extrinsic motivation. This distinction is based on the *orientation* of the motivation, i.e. the type of the motivation. This refers to the underlying reasons, goals and attitudes for the actions and behavior of an individual. ( Ryan & Deci, 2000a)

In SDT, intrinsic motivation is described as “doing something because it is inherently interesting or enjoyable”. Examples are reading about a specific disease because you find the subject genuinely interesting, or playing the piano because you like making music. Intrinsic motivation is a feature inside the individual. People can be intrinsically motivated for one action but for a different action not. Therefore, intrinsic motivation is something that exists in a relation between the individuals and the actions.

Ryan and Deci discuss three primary psychological needs; the need for (a feeling of) autonomy, competence and relatedness. As posed in SDT, these needs must be fulfilled for an individual to get intrinsically motivated for an activity. ( Ryan & Deci, 2000a)

The need for autonomy refers to volition - “ *The organismic desire to self-organize experience and behavior and to have activity be concordant with one’s integrated sense of the self*”. ( Deci & Ryan, 2000) This means that individuals desire that their behavior is the result of their own choice based on a feeling of complete free will. People want their behavior to be an expression of the self. ( Deci & Ryan, 2000; Ten Cate, Kusurkar, & Williams, 2011) The need for competence or effectance refers to - *“A propensity to have an effect on the environment as well as to attain valued outcomes within it”.*( Deci & Ryan, 2000) This indicates that people want to feel effective in whatever activities they perform. It is not about the skill or the ability, but about the feeling and the perception of competence. ( Deci & Ryan, 2000; Ten Cate et al., 2011) The need for relatedness refers to - “ *The desire to feel connected to others - to love and care, and to be loved and cared for*”. ( Deci & Ryan, 2000) Meaning that individuals want to feel connected to other people. This can be achieved, even without a formal membership or relationship. ( Ten Cate et al., 2011)

### Extrinsic motivation

**Figure 1.**
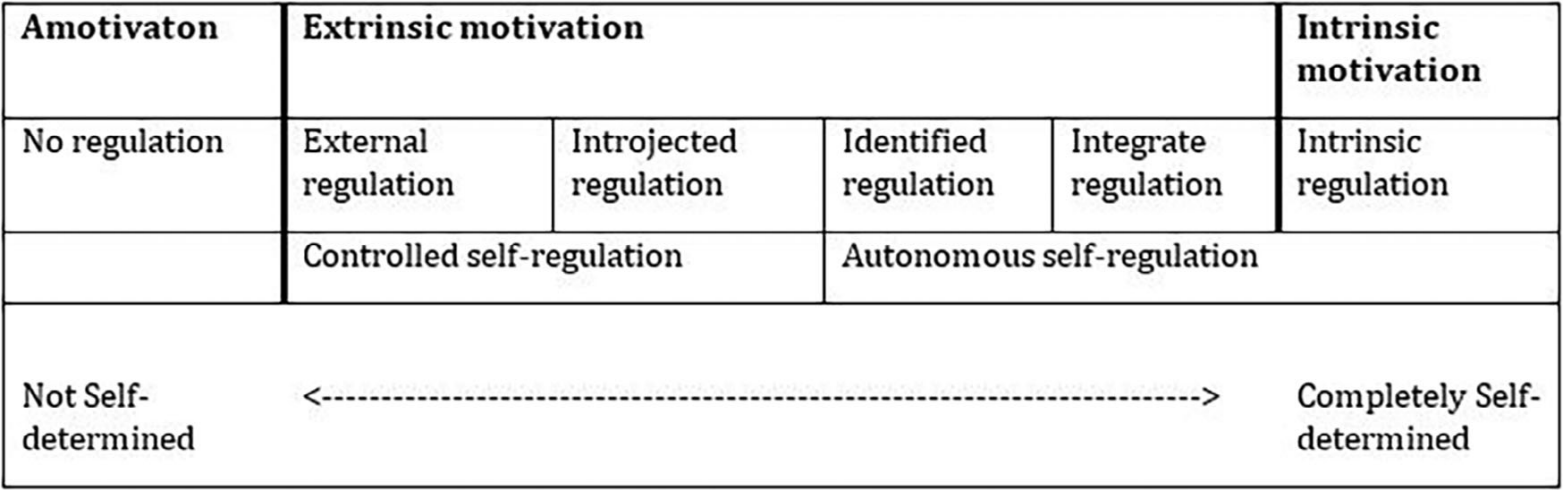
The spectrum of motivation

As many activities are not intrinsically interesting, individuals are often not intrinsically motivated. Individuals are extrinsically motivated when they perform an activity in order to achieve a separable outcome. They are driven based on external factors. ( Ryan & Deci, 2000a; Ten Cate et al., 2011) The SDT poses that the motivation of human behavior can vary on a scale ranging from ‘amotivation’ (lack of motivation), through extrinsic motivation to intrinsic motivation. ( Ryan & Deci, 2000b; Ten Cate et al., 2011) This is displayed in Figure 1.

The feeling of autonomy can vary widely in extrinsically motivated individuals. In SDT, Ryan & Deci discuss the terms *internalization* and *integration.* With these they suggest that individuals can take in values and transform these into their own values. Externally regulated behavior is transformed into a more autonomous self-regulated behavior. This internalization process indicates that the more the activities are integrated by an individual, the more the actions become self-determined. However, extrinsic motivation will always remain extrinsic as the actions serve a separable outcome instead of being satisfying and enjoyable by themselves. ( Legault, 2016; Ryan & Deci, 2000a; Ten Cate et al., 2011) The internalization process is presented as a continuum and describes the range of external motivation from complete external regulation to complete integration. The so-called Orgasmic Integration Theory is a sub-theory of SDT and describes these different forms of extrinsic motivation. It also describes factors that can influence the integration process. ( Ryan & Deci, 2000a; Ten Cate et al., 2011) According to the SDT, the internalization process takes place naturally, without external pressure. ( Ten Cate et al., 2011)

*External regulation* is the category with the least autonomous form of extrinsic motivation. It refers to behavior performed in order to satisfy external demands like deadlines and punishments. Individuals then perceive their behavior as controlled or alienated. The activities have an external perceived locus of causality. ( Legault, 2016; Ryan & Deci, 2000a; Ten Cate et al., 2011)

*Introjected regulation* refers to a type of motivation in which, for instance, rules made by others, are accepted by the individual. People perform actions to avoid guilt or anxiety or to attain pride and self-esteem. This type of motivation still feels quite controlling. ( Legault, 2016; Ryan & Deci, 2000a; Ten Cate et al., 2011)

*Identified regulation* is a more autonomous form of motivation. In this type the individual has identified the personal importance of the actions of behavior. It is a sincere understanding of values of other people or rules made by other people. ( Legault, 2016; Ryan & Deci, 2000a; Ten Cate et al., 2011)

*Integrated regulation* is the most autonomous form of extrinsic motivation. In this case, an individual has internalized the reasons for an action and these regulations has been merged with the self. ( Legault, 2016; Ryan & Deci, 2000a; Ten Cate et al., 2011)

Figure 1 shows this spectrum of motovation schematically, based on representations in the literature ( Ryan & Deci, 2000; Ryan & Deci, 2000b; Ten Cate et al., 2011).

In theory, integrated regulation is distinguishable from intrinsic motivation but in practice, this distinction is less relevant as the individual feels completely autonomous with both types of motivation. ( Ten Cate et al., 2011) As mentioned before, the needs for autonomy, competence and relatedness must be satisfied for an individual to get intrinsically motivated. SDT suggests that these innate psychological needs also play a role in the internalization process of extrinsic motivation. ( Deci, Ryan, & Williams, 1996; Ten Cate et al., 2011)

Effect of external rewards and negative incentives on intrinsic motivation

The level of intrinsic motivation is not static and permanent. There are several conditions that can elicit and enhance, or subdue and diminish the level of intrinsic motivation. The Cognitive Evaluation Theory (CET), another sub-theory of SDT, was developed by Deci and Ryan to specify the factors that influence the intrinsic motivation in individuals. In this theory they argue that individuals must feel competent and autonomous in order to reach a high level of intrinsic motivation. ( Ryan & Deci, 2000a) Research has shown that extrinsic rewards can shift the motivation of individuals from intrinsic to extrinsic as confirmed in a large meta-analysis. ( Deci, Ryan, & Koestner, 1999) This meta-analysis will be discussed more thorough as it is seen as the most important review supporting the CET. ( Ledford, Gerhart, & Fang, 2013)

Ryan and Deci included 128 studies in the meta-analysis to study the effect of extrinsic rewards on intrinsic motivation. Different types of rewards were studied. They started with verbal rewards, i.e. positive feedback, versus tangible rewards. Tangible rewards were divided in those being expected and unexpected ones. Expected tangible rewards were categorized in task-non-contingent rewards, task-contingent rewards and performance-contingent rewards. Task-non-contingent rewards are given for something else than for doing or completing the activity, e.g. for participating in the study. Task-contingent rewards are given for doing or completing the target activity. This reward type can be divided in completion-contingent and engagement-contingent rewards. Completion-contingent rewards are given for completing the task and engagement-contingent rewards are given for engaging in the activity. Performance-contingent rewards are only given after an individual has reached a predetermined level and this type of reward is therefore mostly given to individuals performing the activity well. ( Deci et al., 1999)

In studies investigating intrinsic motivations there are two methods most often used for measuring the level of intrinsic motivation. ( Deci et al., 1999) The first approach is the ‘free-choice’ measure. In this approach, the study participants are exposed to an activity under varying experimental conditions. The participants are given a free-choice period afterwards, in which they can decide to continue the activity or to do something else. ( Deci & Ryan, 2000; Ryan & Deci, 2000a) The second measure is the ‘self-reported’ measure. In this method self-reports are used in which the participants report their interest in and enjoyment of the activity. ( Deci & Ryan, 2000; Ryan & Deci, 2000a) In this meta-analysis, two analyses were performed. One analysis included those studies that used free-choice as a measure for intrinsic motivation and the other analysis included studies that used self-reported interest. ( Deci et al., 1999)

The results show that verbal rewards, i.e. positive feedback, can increase intrinsic motivation in both analyses. Unexpected rewards and task-non-contingent rewards showed no effect on intrinsic motivation. The performance-contingent rewards only had an undermining effect on the intrinsic motivation in the ‘free-choice’ analysis. The other types of rewards undermined the intrinsic motivation on both the ‘free-choice’ and self-reporting analyses. However, the results of the analysis including studies that used self-reported interest as outcome measure were substantially weaker.

This meta-analysis and many other studies focused on the effect of extrinsic rewards on the intrinsic motivation. Fewer studies have investigated the effect of negative extrinsic measurements and incentives like threats, negative feedback, deadlines and competition pressure on the intrinsic motivation. The studies all showed the same results. A very short summary is that these type of measurements and negative incentives also undermine the intrinsic motivation, just like most expected tangible rewards. ( Amabile, DeJong, & Lepper, 1976; Deci & Cascio, 1972; Reeve & Deci, 1996)

In conclusion, expected tangible rewards and also threats, deadlines and competition pressure can diminish intrinsic motivation. An explanation for this effect can be found in the CET. People see themselves as controllers of their own behavior, which, when compromised by external pressure, can diminishes intrinsic motivation. On the other hand, positive feedback increases the feeling of competence and autonomy and with that, the intrinsic motivation. The CET therefore suggests that teachers and parents can improve the intrinsic motivation of an individual by supporting the needs for competence and autonomy. However, it is important to realize that this can only be applied for activities that are enjoyable, interesting or satisfying for that person. ( Ryan & Deci, 2000a)

### How to stimulate motivation in the medical student

So far, we focused on influences of intrinsic motivation. However, according to many studies, the desired type of motivation does not only involve intrinsic motivation. Numerous studies have investigated the effect of autonomous self-regulated learning compared to a more controlled regulation. As displayed in Figure 1, autonomous self-regulated behavior includes intrinsic motivation, integrated regulation and identified regulation. Controlled regulation refers to external and introjected regulation. As summarized in a review by Deci, Ryan & Williams, autonomous self-regulated learning is associated with more positive outcomes compared to a more controlling regulation. For example, students with more autonomous self-regulations experience greater conceptual understanding, perform better and experience enhanced well-being. One study, focused on medical students, showed that students who are more autonomous in their learning felt more competent in interviewing a patient and are stronger in their endorsement of psychosocial values of patients. This indicates that they are more likely to internalize the values that are endorsed in the learning environment. ( Deci et al., 1996)

In conclusion, autonomous self-regulated learning is associated with high learning quality and psychological well-being. In order to increase the motivation of students, teachers could therefore stimulate autonomous self-regulated learning. In SDT literature, this is referred to as autonomy-supportive teaching. Considerable research has showed that satisfying the needs of autonomy, competence and relatedness by the social context can enhance intrinsic motivation and promotes internalization of extrinsic motivation. ( Deci et al., 1996; Ryan & Deci, 2000a; Ten Cate et al., 2011) A study among graduate doctors, who were in training at a neurology department, showed that non-fulfillment of the three basic psychological needs led to dropout or consideration of stopping with the training. ( Van der Linden, 2011) A review published in 2011 and a systematic review published in 2015 investigated how teachers and the teaching environment can support the three basic psychological needs. ( Kusurkar, Ten Cate, Van Asperen, & Croiset, 2011; Orsini, Evans, & Jerez, 2015) In this paper, a classification based on these needs was used to give a summary of practical tips for teachers in health professions which will help to apply autonomy-supportive teaching.

### Enhancing autonomy

The feeling of autonomy will be enhanced in case students feel free to choose. It is therefore important to identify what students want to learn and to give them a choice in what they will learn. ( Orsini et al., 2015) This will make the learning more relevant and can stimulate a genuine interest in the subject. ( Kusurkar, Croiset, & Ten Cate, 2011) Problem-based learning (PBL) is an educational method that gives the students a lot of freedom in the choices they make. ( Kusurkar, Ten Cate, et al., 2011; Lyness et al., 2013; Ten Cate et al., 2011) Students in a PBL curriculum are reportedly more intrinsically motivated compared to students in a traditional curriculum. ( Kusurkar, Ten Cate, et al., 2011) The perceptions of students can also be taken into account by letting them participate in evaluating the success of a curriculum. ( Lyness et al., 2013) Unfortunately, not all activities are interesting and enjoyable for all students and choices cannot always be given. In case the teacher explains the value of the subject for their future profession, the students can internalize the motivation for these activities or subjects. An example is the early introduction of patient contact to show students the value of learning basic science. ( Orsini et al., 2015; Ten Cate et al., 2011) Medical students also feel autonomous and motivated in case the teacher uses different learning approaches. Learning approaches that stimulate students to participate actively are preferred as this enhances students’ achievement and well-being. ( Orsini et al., 2015) The seating arrangement can help to make a lesson more interactive. ( Kusurkar, Croiset, et al., 2011) A teacher could also let the students choose a learning method, task or exercise. Students feel comfortable when they are in charge of their own learning process. Giving students more freedom and providing them choices stimulates the students to take more responsibility which improves their motivation. ( Orsini et al., 2015) A teacher can stimulate students to take more responsibility by giving them extra questions at the end of a lesson that will be discussed in the next session. ( Kusurkar, Croiset, et al., 2011) A really practical advice for teachers to stimulate the autonomous feeling of students is to use words like can, may and could instead of using controlling words like must, should, need. ( Kusurkar, Croiset, et al., 2011; Lyness et al., 2013) At last, it is important to avoid external rewards. As discussed extensively, rewards can undermine the intrinsic motivation. ( Deci et al., 1999; Orsini et al., 2015) Students who are autonomously motivated will prepare and will participate in class. It would be better to let this internal state guide the behavior of the students instead of providing or withholding rewards. ( Kusurkar, Croiset, et al., 2011)

### Enhancing competence

In order to support the feeling of competence, the teacher could provide optimal, ‘just-right’ challenges. This means that the challenges should not be too hard and not too easy. ( Lyness et al., 2013) An option could be to let the student prepare topics in groups and let them present their part. This can help the students to feel competent in their learning as they will also explain their results to the other students. ( Kusurkar, Croiset, et al., 2011) In adjustment to that, it is helpful to provide the right combination of experiences and conditions to enable the students to develop the required skills. This means that skills training should connect to the level of the challenges and the required skills. ( Lyness et al., 2013) Next, is important to value the work the students do. A relatively easy way to do this, is to give positive and constructive feedback to the student. The feedback should be focused on the task and not on the student. ( Orsini et al., 2015) Pendleton’s rules of feedback are often used in medical education to provide feedback. ( Chowdhury & Kalu, 2004) Positive feedback does not mean that feedback on errors cannot be given, but that these points should be phrased as ‘points of improvement’ which gives it a less negative tone. ( Kusurkar, Croiset, et al., 2011; Lyness et al., 2013) At last, it is important to provide structured guidance, as autonomous self-regulated learning does not mean that the students are completely dependent on their own. The teacher is there to guide the students in the right direction, to structure the session and to correct them in case they go in the wrong direction. ( Kusurkar, Croiset, et al., 2011) Previous tips can all be applied by teachers. The review of Kusurkar et al. also mentions aspects that can be taken into account by academic leaders. First, students who enter medical school through a selection procedure show a higher motivation compared to students entering through lottery or high grades in high school. ( Kusurkar, Ten Cate, et al., 2011) Curriculum planners can consider starting a selection procedure. The type of assessment can also influence the feeling of competence. Comparison with pre-set standards and meeting these standards can stimulate the feeling of competence in medical students. On the opposite, students who are assessed by a comparison with peers can start feeling incompetent, even if this is not justified. Finally, results show that an honours system can substantially demotivate students. Students who know they will not get honours, might feel incompetent. ( Kusurkar, Ten Cate, et al., 2011)

### Enhancing relatedness

To achieve autonomous self-regulated behavior in students, it is important that they feel related to teachers, parents, peers and also patients. ( Kusurkar, Ten Cate, et al., 2011; Orsini et al., 2015) Students with strong feelings of relatedness with family and colleagues reportedly have a higher motivation. ( Kusurkar, Ten Cate, et al., 2011) Multiple studies showed that the teacher’s personal qualities are important in motivating the students. Motivating qualities are; encouraging, open-mindedness, creative, accessible, happy, interesting, promoting class discussion, approachability, concern for students and fairness. These qualities can be difficult to achieve in a short time. Relatively easy actions for teachers, helping them to improve the feeling of relatedness of students, is to respect the students and to give them emotional support. It is important for students to feel part of the team. They also want to feel free to express their opinion. This is often referred to as a safe learning environment in which students feel free to ask questions, express their doubts and to share their feelings. Further, an autonomy-supportive teacher should be empathetic, also when the students criticize the work of the teacher. When teachers become defensive instead of listening and discussing, students will become demotivated. It is important that the students feel heard. ( Orsini et al., 2015) Finally, academic leaders can enhance the feeling of relatedness by facilitating the formation of individual connections. In small group teaching students will develop more personalized connections than in lectures to a big group of students. On the other hand, it is also important that the students feel connected to the group as a whole. This can be stimulated with both curricular and extracurricular large-group activities. ( Lyness et al., 2013)

## Conclusion

Teachers often apply extrinsic rewards or punishments to improve the motivation of the students. The main aim of this paper was to study the effect of rewards and negative incentives on the intrinsic motivation of medical students. In this paper, the most important meta-analysis on this subject was discussed. The results showed that verbal rewards can improve the intrinsic motivation.

Task-non-contingent rewards and unexpected tangible rewards appear to have no distinct effect on the intrinsic motivation. All other types of expected tangible rewards undermine the intrinsic motivation. Other studies show that negative incentives like threats, deadlines and competitive pressure also reduce intrinsic motivation. ( Amabile et al., 1976; Deci & Cascio, 1972; Deci et al., 1999; Reeve & Deci, 1996) Therefore, extrinsic rewards and negative incentives are not effective methods to try increasing motivation in medical students.

Multiple studies have confirmed that autonomous self-regulated learning, including intrinsic motivation, identified regulation and/or integrated regulation, results in high quality learning and well-being compared to more controlled learning. This indicates that, to improve the motivation of medical students, teachers can be advised to focus on autonomous types of motivation. This is referred to as *autonomy-supportive teaching* in which the satisfaction of the three basic psychological needs is stimulated. ( Deci et al., 1996; Ryan & Deci, 2000a; Ten Cate et al., 2011) Several practical tips that all support one of the three basic psychological needs were discussed. These tips are relatively easy to apply as a teacher and will help to improve the intrinsic motivation or will stimulate internalization of extrinsic motivation in medical students.

## Take Home Messages

Extrinsic rewards and negative incentives should be avoided as they both undermine the intrinsic motivation. Autonomous self-regulated learning leads to more effective learning. Several practical tips that support one of the three basic psychological needs are discussed. Most are relatively easy to apply and stimulate autonomous self-regulated learning.

## Notes On Contributors

First author Nienke Henson is a medical student at UMC Utrecht, the Netherlands and wrote the first version of this paper during a Student Teaching Rotation.

Senior author Olle ten Cate is Professor of Medical Education at UMC Utrecht, the Netherlands and advised on the paper and edited text for intellectual content.
