# Supplementary material for: How to deal with the unmotivated medical student in small group sessions?
Source: MedEdPublish (2016). 2017 May 19;6:86. [Version 1] doi: 10.15694/mep.2017.000086 (PMC10885256; doi:10.15694/mep.2017.000086)
Supplement: Peer review history

# Reviewer report: Reviewer response for version 1

**DOI:** 10.21956/mep.19300.r28132

**Published:** 2017-06-01

**Authors:** Susmita Reddy Karri

**Affiliations:** 1 Topiwala national medical college and BYL nair charitable hospital, mumbai. india

This review has been migrated. The reviewer awarded 3 stars out of 5 Being a medical student myself, I totally agree that teaching in small groups definitely helps and the enthusiasm and dedication of the educator plays a major role in moulding a students' career and interest.However,I would like to know more about the 'Intrinsic rewards.'Also, I would have loved if this paper was more focused on implementing/overcoming difficulties rather than just listing them.

Reviewer Expertise:

NA

No decision status is available

# Reviewer report: Reviewer response for version 1

**DOI:** 10.21956/mep.19300.r28125

**Published:** 2017-05-24

**Authors:** BALAJI ARUMUGAM

**Affiliations:** 1 TAGORE MEDICAL COLLEGE AND HOSPITAL

This review has been migrated. The reviewer awarded 4 stars out of 5 The entire article was so interesting and its an eye opener for the new and very old medical teachers. As a reader and medical teacher I felt that this particular sentence in conclusion part "Extrinsic rewards and negative incentives should be avoided as they both undermine the intrinsic motivation", is to be noted. But for any facilitator avoiding the extrinsic rewards is the most difficult to be practiced. I personally feel that this article is well organized and need of the hour for many countries where medical education technologies are still in rudimentary stage.

Reviewer Expertise:

NA

No decision status is available

# Reviewer report: Reviewer response for version 1

**DOI:** 10.21956/mep.19300.r28126

**Published:** 2017-05-22

**Authors:** Tan Nguyen

**Affiliations:** 1 Deakin University

This review has been migrated. The reviewer awarded 2 stars out of 5 This paper provides a general overview on the literature surrounding self-determination theory. It provides a good foundation knowledge in the field. However, this review lacks significant details of a systematic review of research rigour. It would be beneficial to refer to the systematic review methodology by Bearman and colleagues (2012) or similar. As a result, there are implied conclusions or conjectures made, rather than justified conclusions. The included papers should be subjected to quality appraisal.Bearman, M., Smith, C. D., Carbone, A., Slade, S., Baik, C., Hughes-Warrington, M., & Neumann, D. L. (2012). Systematic review methodology in higher education. Higher Education Research & Development, 31(5), 625-640.

Reviewer Expertise:

NA

No decision status is available

# Reviewer report: Reviewer response for version 1

**DOI:** 10.21956/mep.19300.r28127

**Published:** 2017-05-21

**Authors:** Susan Van Schalkwyk

**Affiliations:** 1 Stellenbosch University

This review has been migrated. The reviewer awarded 3 stars out of 5 I was attracted by the title of this article and based on the number of reviews it has already garnered, I suspect that others were too. The article provides a detailed theoretical account of how motivation influences the way in which students engage in small group work. Although I found the article interesting, I did wonder about the claim made that teachers ‘apply extrinsic awards or punishments’ to enhance student motivation – this probably deserves further consideration. I also wondered why the authors opted to only make use of Google Scholar and how the ‘most relevant’ articles were selected. It would have been interesting to have had this insight into the methodology followed. Nevertheless, the findings of the study point to issues of autonomy, competence and relatedness and reminds us of how critical it is for teachers to be cognisant of the role that these factors can play in enhancing student engagement.

Reviewer Expertise:

NA

No decision status is available

# Reviewer report: Reviewer response for version 1

**DOI:** 10.21956/mep.19300.r28130

**Published:** 2017-05-20

**Authors:** Subha Ramani

**Affiliations:** 1 Harvard Medical School, Brigham and Women's Hospital

This review has been migrated. The reviewer awarded 4 stars out of 5 I think the self-determination theory is very important to educators who design strategies to motivate learners. It also fits in with the adult learning theory which emphasizes self-directed learning, relevance etc. This paper is a good summary of the theory and strategies to enhance autonomy, relatedness and competence, It also cautions educators that negative Incentives can be detrimental to motivation. As expressed by other reviewers, I am eager to learn about specific methods to enhance internal motivation and how it can impact deep learning and critical thinking.Overall I enjoyed reviewing the tenets of SDT and look forward to more studies in this area.Would have liked more specifics on strategies that fall within positive and negative incentives.Good review of the theory, it principles and practice.

Reviewer Expertise:

NA

No decision status is available

# Reviewer report: Reviewer response for version 1

**DOI:** 10.21956/mep.19300.r28131

**Published:** 2017-05-19

**Authors:** P Ravi Shankar

**Affiliations:** 1 American International Medical University

This review has been migrated. The reviewer awarded 3 stars out of 5 I enjoyed reading this interesting paper. I often use small group, active learning methods in the classroom. In Nepal with large class sizes of 100 students or more, group sizes were large and my fellow faculty members helped as co-facilitators. In the Caribbean schools in Aruba (where I had worked) and Saint Lucia (where I am presently working), class sizes are small and there is a greater interaction between students and faculty, facilitating small group learning. The scenario mentioned by the authors at the beginning of the paper is a familiar one to all educators.It has been my experience that in each cohort of students, there are some willing to assume greater responsibility for their own learning while others are reluctant. The authors provide a detailed description of extrinsic, intrinsic motivation and the self-determination theory though occasionally I found their arguments somewhat difficult to understand. The section on how to stimulate motivation in the medical student was interesting in a general sense. A problem I have often faced is in a group, one or two students may not be motivated enough and can bring down the group performance. I would have liked advice on how to specifically motivate these students. Sometimes, specific members of a group have poor interpersonal relations. I agree with the reviewer, Dr Cookson on how little choice medical curricula offers students on what to learn and how to learn it though the situation is improving. Relatedness and a sense of belonging are very important. In my many years as a medical educator, I have noted that some cohorts of students have a greater sense of relatedness and perform better than other groups, where it was every student by and for himself or herself.  

Reviewer Expertise:

NA

No decision status is available

# Reviewer report: Reviewer response for version 1

**DOI:** 10.21956/mep.19300.r28128

**Published:** 2017-05-19

**Authors:** Julie Hunt

**Affiliations:** 1 Lincoln Memorial University

This review has been migrated. The reviewer awarded 4 stars out of 5 This article is a great introduction to learning theory and uses a meta analysis to answer, in broad terms, a question that many educators find themselves asking: how do I get my students to want to learn? This article was stronger in describing theory than it was in describing implementation, The tips it does offer are somewhat vague, which is both a weakness and a strength. More specific recommendations may be more helpful to some educators, but the less specific recommendations could be adapted to almost any medical learning environment. I would love to also see an expanded version of this paper which reviews the implementation methods in more detail. As it is now, it is a good manuscript for introducing self-determination theory and how motivation can be encouraged or discouraged in the learning environment.

Reviewer Expertise:

NA

No decision status is available

# Reviewer report: Reviewer response for version 1

**DOI:** 10.21956/mep.19300.r28123

**Published:** 2017-05-19

**Authors:** Shelley Fielden

**Affiliations:** 1 Leeds Institute of Medical Education

This review has been migrated. The reviewer awarded 4 stars out of 5 This article will be of particular interest to students and staff involved in formal teaching who have some influence over how material is delivered and the setting in which it is taught. The authors present a number of helpful tips to increase learner engagement and I particularly welcomed the discussion about how to engage learners in what they wish to learn in order to make the most from the formal teaching setting. This approach could include asking students to identify learning needs at the start of a taught session and tailoring the material accordingly with curricula becoming personalised to the particular learning needs of individual students. I would like to have seen some discussion of the challenges in adopting this approach (e.g. responding to differing individual needs of a large student group, need for facilitators to be flexible in approach and to facilitate rather than teach etc.). The paper is well written and makes a powerful case for looking at how we as educators better engage our students in learning beyond the carrot and stick approach.

Reviewer Expertise:

NA

No decision status is available

# Reviewer report: Reviewer response for version 1

**DOI:** 10.21956/mep.19300.r28129

**Published:** 2017-05-19

**Authors:** Trevor Gibbs

**Affiliations:** 1 AMEE

This review has been migrated. The reviewer awarded 3 stars out of 5 An interesting paper to read that explored a very interesting and important area of medical education related to student motivation.I was initially confused since from the title I was expecting a paper describing an activity within a school that attempted to demonstrate how an activity had increased student motivation, in small groups.However, the paper seemed to be a mixture of explaining the theory(ies) behind motivation and a review of some of the papers written.However, I still enjoyed reading it and I though the authors explained the nuances of intrinsic and extrinsic motivation very well. I was surprised that the authors only used Google Scholar for their search but important papers did result.Like my co-reviewer and I think like many who read this paper, I was looking for more answers to implementation rather than vague suggestions, and the difficulties to be overcome in implementation.I also missed the discussion around how faculty motivation affects the students, given I think that internal motivation is often driven or increased by external human forcesA useful paper to read for those involved in the area

Reviewer Expertise:

NA

No decision status is available

# Reviewer report: Reviewer response for version 1

**DOI:** 10.21956/mep.19300.r28124

**Published:** 2017-05-19

**Authors:** John Cookson

**Affiliations:** 1 University of Worcester

This review has been migrated. The reviewer awarded 5 stars out of 5 I enjoyed this very much and there are some important lessons about motivation for anyone who teaches medical students; in particular, that applying external motivation (rewards and punishments) does not work. It left me reflecting on how badly served our students and junior doctors can be sometimes. Autonomy, competence and relatedness are the three important psychological needs identified here but what have we done in recent years to enhance these? Curricula have been more closely defined with reduction in student choice. The patient safety agenda, important though it is, leaves students in a Catch 22; they are not competent because they can’t practice and they can’t practice because they are not competent. As for relatedness, the teams as firms have been abolished, doctors’ messes closed and shift work means constant change of colleagues.

Reviewer Expertise:

NA

No decision status is available
